# Upregulation of Heme Oxygenase-1 by Hemin Alleviates Sepsis-Induced Muscle Wasting in Mice

**DOI:** 10.1155/2018/8927104

**Published:** 2018-11-08

**Authors:** Xiongwei Yu, Wenjun Han, Changli Wang, Daming Sui, Jinjun Bian, Lulong Bo, Xiaoming Deng

**Affiliations:** ^1^Faculty of Anesthesiology, Changhai Hospital, Naval Medical University, Shanghai 200433, China; ^2^Department of Anesthesiology, 285th Hospital of the CPLA, Handan 056001, China; ^3^Department of Anesthesiology, Chengdu Military General Hospital, Chengdu 610083, China

## Abstract

Hemin, an inducer of heme oxygenase-1 (HO-1), can enhance the activation of HO-1. HO-1 exhibits a variety of activities, such as anti-inflammatory, antioxidative, and antiapoptotic functions. The objective of this study was to investigate the effects of hemin on sepsis-induced skeletal muscle wasting and to explore the mechanisms by which hemin exerts its effects. Cecal ligation and perforation (CLP) was performed to create a sepsis mouse model. Mice were randomly divided into four groups: control, CLP, CLP plus group, and CLP-hemin-ZnPP (a HO-1 inhibitor). The weight of the solei from the mice was measured, and histopathology was examined. Cytokines were measured by enzyme-linked immunosorbent assay (ELISA). Real-time quantitative reverse transcription polymerase chain reaction (qRT-PCR) and Western blotting were used to assess the expression levels of HO-1 and atrogin-1. Furthermore, we investigated the antioxidative effects of HO-1 by detecting malondialdehyde (MDA) levels and superoxide dismutase (SOD) activity. CLP led to dramatic skeletal muscle weakness and atrophy, but pretreatment with hemin protected mice against CLP-mediated muscle atrophy. Hemin also induced high HO-1 expression, which resulted in suppressed proinflammatory cytokine and reactive oxygen species (ROS) production. The expression of MuRF1 and atrogin-1, two ubiquitin ligases of the ubiquitin-proteasome system- (UPS-) mediated proteolysis, was also inhibited by increased HO-1 levels. Hemin-mediated increases in HO-1 expression exert protective effects on sepsis-induced skeletal muscle atrophy at least partly by inhibiting the expression of proinflammatory cytokines, UPS-mediated proteolysis, and ROS activation. Therefore, hemin might be a new treatment target against sepsis-induced skeletal muscle atrophy.

## 1. Introduction

Sepsis is defined as a life-threatening organ dysfunction due to a dysregulated host response to infection [1]. In the United States, nearly 10% of all deaths result from severe sepsis or its related complications every year [2]. Skeletal muscle atrophy and muscle weakness occurring from sepsis have become recognized as important issues in sepsis survivors [3]. A large number of critically ICU patients suffer from severe muscle wasting and impaired muscle function, which can delay respirator weaning and persist long after hospital discharge, thus reducing the patients' quality of life [4, 5].

Muscle atrophy results from an imbalance between muscle proteolysis and protein synthesis. When proteolysis overwhelms protein synthesis, muscle atrophy occurs [6, 7]. Protein degradation within muscle appears to rely on three pathways: ubiquitin-proteasome system- (UPS-) mediated proteolysis, autophagy, and calcium-dependent calpains [8]. However, the pathway that has received the most attention is the UPS-mediated proteolysis, which is believed to play a dominant role in skeletal muscle atrophy [9]. Two ubiquitin ligases, MuRF1 and atrogin-1, are key positive regulators of UPS-mediated proteolysis and are upregulated in all rodent models of skeletal muscle atrophy [10–12]. Additionally, these proteins have been widely used as markers of muscle wasting. Sepsis-induced cytokine secretion can also enhance microvascular permeability, allowing circulating toxins to impair axon activity [13]. The nutrition deficiency in muscle caused by impaired axons may lead to muscle atrophy. As some myofibrillar proteins possess sulfhydryl groups that are sensitive to oxidation, sepsis-induced reactive oxygen species (ROS) appear to contribute to muscle wasting [14]. Thus, inhibiting proinflammatory cytokines and ROS should be an effective method to reverse muscle wasting.

Heme oxygenase-1 (HO-1), also called heat shock protein 32 (Hsp32), is an inducible enzyme that can convert heme into carbon monoxide, biliverdin, and free iron [15, 16]. Recent findings reported that HO-1 and its metabolites exerted anti-inflammatory, antioxidative, and antiapoptotic activities [17, 18]. As metabolites of HO-1, CO and biliverdin were shown to contribute to stimulating the host defense response against sepsis and modulating inflammatory mediators in mice [18]. Previous studies support the beneficial effects of HO-1 and its product in an experimental model of sepsis [19]. We hypothesized that the induction of HO-1 plays a pivotal role in sepsis-induced skeletal muscle wasting.

In our study, we used hemin as an inducer of HO-1 and examined whether hemin exerts a protective effect against septic muscle atrophy in mice. We also investigated the potential mechanism of its protective effect.

## 2. Materials and Methods

### 2.1. Sepsis Model

Cecal ligation and perforation (CLP) was performed on 8-week-old male C57BL/6 mice obtained from the Experimental Animal Center of the Naval Medical University. All animals were fed a standard laboratory diet and water and were acclimatized for at least l week before use. All experimental procedures involving animals were approved by the Animal Care and Use Committee of the Second Military Medical University. After we anesthetized the mice with 2%–3% sevoflurane, a midline laparotomy was performed, and the cecum was exposed. The contents of the intestines were extruded to the tip of the cecum, and then the cecum was ligated 1 cm from the tip with a 3-0 silk suture. We performed a double puncture of the cecum wall with a 22-gauge needle. The abdominal wall was closed with a continuous 3-0 silk suture in two layers. Sham-operated mice were subjected to exposure of the peritoneum and cecum but did not undergo ligation or puncture. No antibiotics were used. The mortality of the septic mice is 25% at day 1, while it elevated to 50% at day 7, indicating that the severity is moderate to severe.

### 2.2. Chemical Treatment and Experimental Groups

Hemin and zinc protoporphyrin-IX (ZnPP, a HO-1 inhibitor) (Sigma-Aldrich, St. Louis, MO) were dissolved in dimethyl sulfoxide (DMSO) and then diluted with phosphate-buffered saline (PBS) to a concentration of 10 mg/ml. Mice were randomly divided into four groups: control group (A), CLP group (B), CLP plus hemin group (C), and CLP-hemin-ZnPP group (D). Mice in groups A and B were intraperitoneally (i.p.) injected with 200 *μ*l PBS containing 2% DMSO 1 day before surgery. Mice in group C were injected with the same volume of hemin solution (50 mg/kg, i.p.) 1 day before surgery, and mice in group D were injected with the same volume of a mixture of hemin (50 mg/kg, i.p.) and ZnPP (20 mg/kg, i.p.). We provided the same quantity of food and water to all four groups.

### 2.3. Measurements of Muscle Mass and Protein Breakdown Rates

The skin of the hind limbs was stripped for gross comparisons of muscle mass. The solei of the mice were collected and weighed at baseline and at 1, 3, 5, and 7 days after surgery. We interpreted the weight of soleus as an evaluation of muscle mass loss. Twenty-four hours after CLP or sham surgery, the solei of the mice were dissected and incubated for 2 hours in a shaking water bath at 37°C as previously described [20, 21]. Because tyrosine cannot be synthesized or degraded in muscle, protein breakdown rates were determined by testing the net release of free tyrosine into the incubation medium containing cycloheximide, a compound that prevents the reincorporation of tyrosine into protein.

### 2.4. Histopathology Examination

Tibialis anterior (TA) tissues were fixed with 10% buffered formalin at room temperature, embedded in paraffin, and sliced into 5 *μ*m sections. The sections were then stained with hematoxylin-eosin (H&E) for morphological evaluation under a light microscope (Leica, DM-IL-LED). We observed the transverse sections taken at the midpoints of the legs. The muscle fiber size was recorded by measuring the cross-sectional area (CSA) of the skeletal muscle fiber.

### 2.5. Real-Time Quantitative Reverse Transcription Polymerase Chain Reaction (qRT-PCR)

Six mice from each group were randomly killed 1, 4, and 7 days after CLP, after which the TA muscles were harvested. Total RNA was extracted from the TA tissue using TRIzol reagent (Invitrogen) according to the manufacturer's instructions. cDNA was synthesized using a PrimeScript™ 1^st^ strand cDNA synthesis kit (TaKaRa). Quantitative PCR was performed using a SYBR green PCR kit (Invitrogen, Carlsbad, CA). The PCR conditions were as follows: initial heating at 95°C for 30 s, 40 cycles of denaturation at 95°C for 5 s and annealing at 60°C for 30 s, one extension at 95°C for 15 s, and a final extension at 60°C for 30 s. The following primers were used: 5′-TGTGGGTGTATCGGATGGAG-3′ and 5′-GGCAGAGTCTTCCACAGT-3′ for atrogin-1, 5′-TTTGACACCCTCTACGCCAT-3′ and 5′-TTGGCACTTGAGAGAGAGGAAGGT-3′ for MuRF1, and 5′-TTCAGAAGGGTCAGGTCC-3′ and 5′-CAGTGAGGCCCATACCAGAA-3′ for HO-1.

The generation of PCR products was identified by melting-curve analysis. Relative mRNA levels of atrogin-1, MuRF1, and HO-1 were normalized to those of GAPDH.

### 2.6. HO-1 Activity Assay

The activity of HO-1 was identified via bilirubin generation according to the method described by Gong et al. [18]. Briefly, frozen TA samples were homogenized in lysis buffer. A sample fraction was harvested and washed by multiple centrifugations. The pellet was solubilized in 0.1 M K_2_HPO_3_ by sonication and stored at −80°C until extraction with chloroform. The extracted bilirubin was measured by the difference in absorbance at 464 nm and 530 nm.

### 2.7. Cytokine Analysis

Blood was collected via cardiac puncture with heparin-treated syringe needles and centrifuged at 1000*g* for 10 min to harvest the serum. Sera were assayed for mouse TNF-*α* and IL-6 with a murine enzyme-linked immunosorbent assay (ELISA) kit (eBioscience, San Diego, CA) as described by the manufacturer.

### 2.8. Western Blotting Analysis

Briefly, the TA tissues were homogenized, and the proteins were resolved on polyacrylamide SDS gels and electrophoretically transferred to polyvinylidene difluoride membranes. The membranes were blocked with 5% (*w*/*v*) fat-free milk in Tris-buffered saline containing 0.05% Tween-20, incubated with Abs (Abcam) against mouse HO-1, atrogin-1, or MuRF1 at 4°C overnight, and finally incubated with secondary Abs. After the immunoreactive protein bands were visualized, the protein levels were normalized to the band density of *β*-actin (Abcam), which served as an internal control. The corresponding semiquantitative analysis was performed by measuring the optical density using the ImageJ software, and *β*-actin was used as an internal control.

### 2.9. Assay of TA Lipid Peroxidation and Antioxidant Enzyme Activities

Malondialdehyde (MDA), the end product of lipid peroxidation, is a marker of tissue peroxidation. We evaluated the degree of tissue peroxidation by measuring the MDA levels. Superoxide dismutase (SOD) activity was measured to analyze the activity of antioxidant enzymes. At 24 hours after CLP, we harvested the TA muscle and measured the MDA levels and SOD activity according the methods described by Andrianjafiniony et al. [22].

### 2.10. Statistical Analysis

The study results are presented as the mean ± standard deviation (SD). Differences between group means were calculated by one-way analysis of variance (ANOVA) or Student's *t*-test. All data were analyzed using Prism 5.0 (GraphPad Software, USA). *p* < 0.05 was considered statistically significant.

## 3. Results

### 3.1. Administration of Hemin Ameliorated the Loss of Muscle Mass

**Figure 1:**
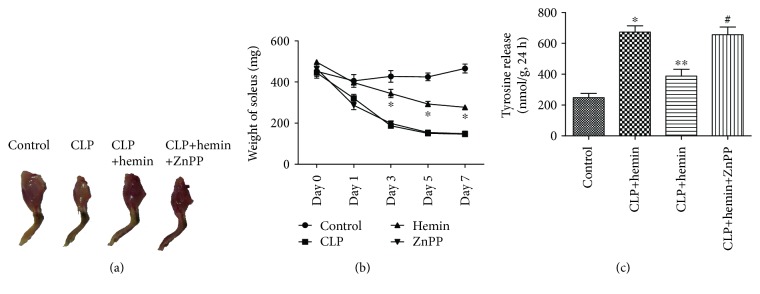
Hemin ameliorated muscle mass loss and mitigated the protein breakdown rates. (a) Gross comparisons of muscle mass (8-week-old mice, 3 days after CLP surgery). (b) The weight of the solei from mice in the hemin group was improved, especially on days 3, 5, and 7 after surgery. ^∗^*p* < 0.001 (hemin vs. CLP), *n* = 6. (c) Protein breakdown rates as measured by the tyrosine concentration. ^∗^*p* < 0.001 (control vs. CLP), ^∗∗^*p* < 0.001 (hemin vs. CLP), ^#^*p* < 0.001 (ZnPP vs. hemin), *n* = 6.

As shown in Figure 1(a), the size of the hind limbs was significantly atrophic after CLP surgery. However, administration of hemin partly reduced this effect, but treatment with ZnPP, an inhibitor of HO-1, reversed the effects of hemin. The weight of the solei among the groups was not significantly different at baseline. As the time after surgery increased, only slight soleus weight loss was observed in the control group. However, there was a significant deterioration in the weight of the solei from septic mice. Administration of hemin ameliorated this loss in muscle mass, while ZnPP abrogated this protective effect (Figure 1(b)). These results suggested that hemin exerts a protective effect against the loss of muscle mass. Protein breakdown rates in septic mice increased by nearly 2-fold. There was no significant difference in the protein breakdown rates between the control and hemin groups, and the protein breakdown rates in the ZnPP group were similar to those in the CLP group (Figure 1(c)).

### 3.2. Hemin Pretreatment Improved Sepsis-Induced Pathological Injury of TA Tissues of Mice

**Figure 2:**
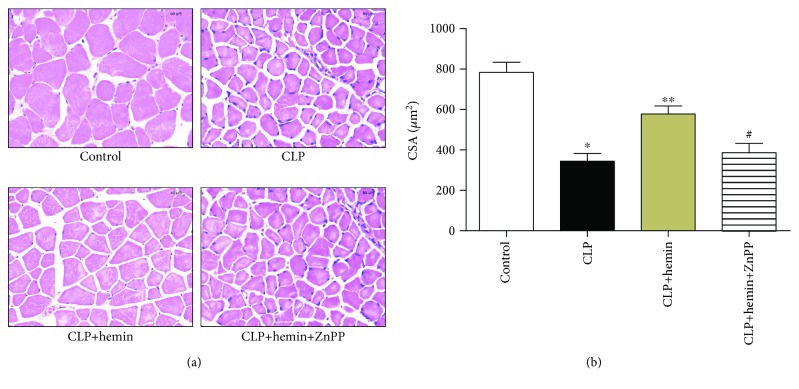
Histopathology examination of TA muscles at day 5 after surgery. (a) Skeletal muscle fibers from the CLP group exhibited a smaller cross-sectional area (CSA) than those from the control and hemin groups (H&E staining, 40x magnification, scale bar = 50 *μ*m). (b) Quantification of the CSA of TA muscles from the different groups. ^∗^*p* < 0.001 (CLP vs. the control group), ^∗∗^*p* < 0.01 (hemin vs. the CLP group), ^#^*p* < 0.05 (ZnPP vs. the hemin group), *n* = 6.

The TA tissues from the CLP group revealed smaller myofibers and CSA; however, the muscle fibers from the hemin group were in better condition than those from the CLP group (Figure 2(a)). We used the CSA of the muscle fibers to assess the degree of muscle atrophy. The CSA of the fibers from the CLP group was significantly smaller than that of the fibers from the control group. The administration of hemin positively affected the myofiber size, whereas mice in the ZnPP group showed a similar myofiber size to mice in the CLP group (Figure 2(b)).

### 3.3. Hemin Upregulated the Expression and Activity of HO-1 in TA

**Figure 3:**
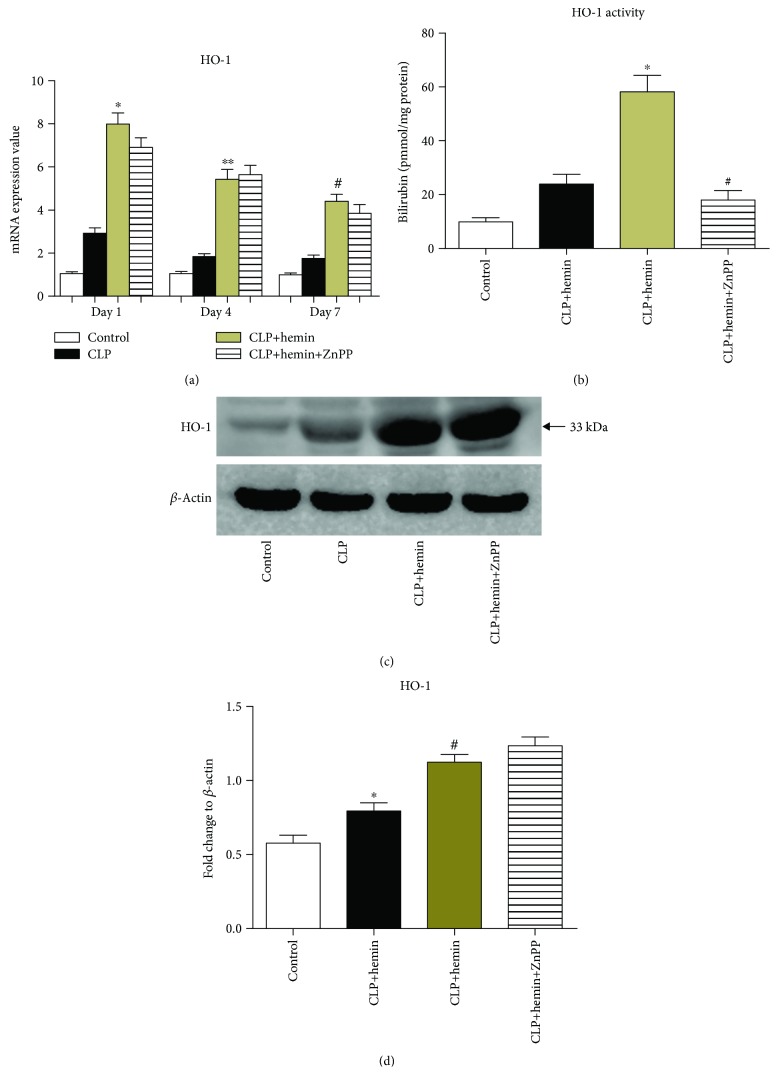
Hemin induces high HO-1 expression and increased HO-1 activation. (a) Hemin pretreatment can promote HO-1 mRNA expression at 1, 4, and 7 days after surgery (^∗^*p* < 0.001 vs. CLP at 1 day, ^∗∗^*p* < 0.001 vs. CLP at 4 days, ^#^*p* < 0.05 vs. CLP at 7 days). (b) Hemin enhanced the enzyme activity of HO-1, but ZnPP treatment reversed this effect (^∗^*p* < 0.01 vs. CLP, ^#^*p* < 0.01 vs. CLP + hemin; *n* = 6). (c) Western blot analysis of levels of the HO-1 protein. (d) Results of the corresponding semiquantitative analysis of levels of the HO-1 protein based on the optical density measured using the ImageJ software; the data are presented as means ± SEM and are representative of three separate experiments (^∗^*p* < 0.01 vs. control, ^#^*p* < 0.01 vs. CLP).

The qRT-PCR results showed that HO-1 levels and activity were higher in the CLP group than in the sham group (*p* < 0.05), indicating that sepsis could induce HO-1 activation and its activity. We also found that hemin administration significantly increased the HO-1 levels and its activation and that the change in HO-1 expression in the hemin group was higher than that in the control and CLP groups (Figure 3(a)). ZnPP, an inhibitor of HO-1, suppressed HO-1 activation but did not affect hemin-induced upregulation of HO-1 expression (Figure 3(b)). We also found that the protein expression of HO-1 was slightly elevated in the CLP group compared with that in the control group. Pretreatment with hemin significantly enhanced HO-1 protein expression, but ZnPP administration did not affect the increased expression of HO-1 (Figures 3(c) and 3(d)).

### 3.4. Hemin Contributed to the Reduction in Proinflammatory Cytokine Levels

**Figure 4:**
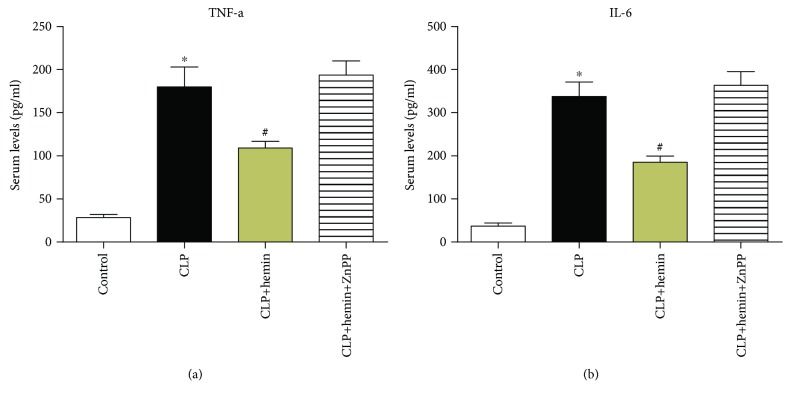
Sepsis induced high serum levels of TNF-*α* and IL-6. Hemin pretreatment reduced the levels of TNF-*α* and IL-6. (a) ^∗^*p* < 0.0001 vs. control, ^#^*p* < 0.05 vs. CLP; *n* = 6. (b) ^∗^*p* < 0.0001 vs. control, ^#^*p* < 0.01 vs. CLP; *n* = 6 (1 day after surgery).

Because changes in proinflammatory cytokine levels play an important role in sepsis and may be involved in muscle atrophy, we examined the production of cytokines. The plasma levels of the proinflammatory cytokines TNF-*α* and IL-6 were significantly upregulated in mice that underwent the CLP procedure compared with those in mice from the sham group (Figure 4). High HO-1 expression induced by hemin could suppress these increases, and the serum levels of TNF-*α* and IL-6 did not show a significant difference between the CLP and ZnPP groups.

### 3.5. Hemin Enhanced the Antioxidant Defense Response

**Figure 5:**
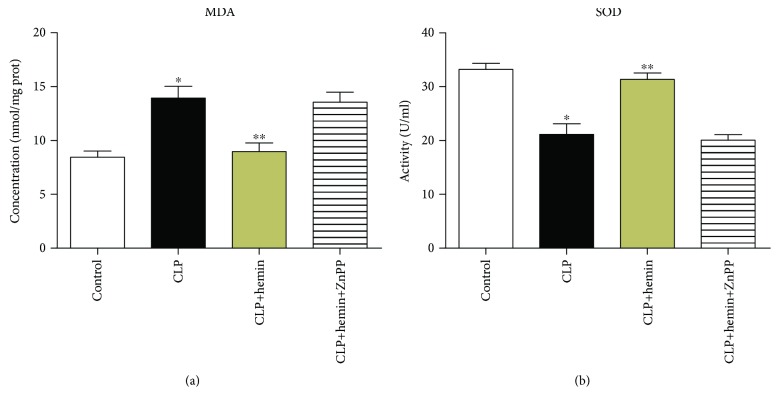
Oxidative stress response in TA muscle at 24 hours after CLP. (a) MDA concentration. ^∗^*p* < 0.05 vs. the control group; ^∗∗^*p* < 0.05 vs. the CLP group; *n* = 6. (b) SOD activity measurement. ^∗^*p* < 0.01 vs. the control group; ^∗∗^*p* < 0.01 vs. the CLP group; *n* = 6.

Compared with the control group, the CLP group showed a statistically significant increase in the plasma levels of MDA. MDA levels were lower in the hemin-pretreated mice than in mice in the CLP group. MDA levels in mice in the ZnPP group were slightly higher than those in mice in the CLP group, but there was no statistically significant difference (Figure 5(a)). Muscle SOD activity in mice in the hemin group was obviously higher than that in mice in the CLP group, and the protective function of hemin was abrogated by ZnPP (Figure 5(b)).

### 3.6. Hemin Inhibited the Expression of Muscle Atrophy Markers

**Figure 6:**
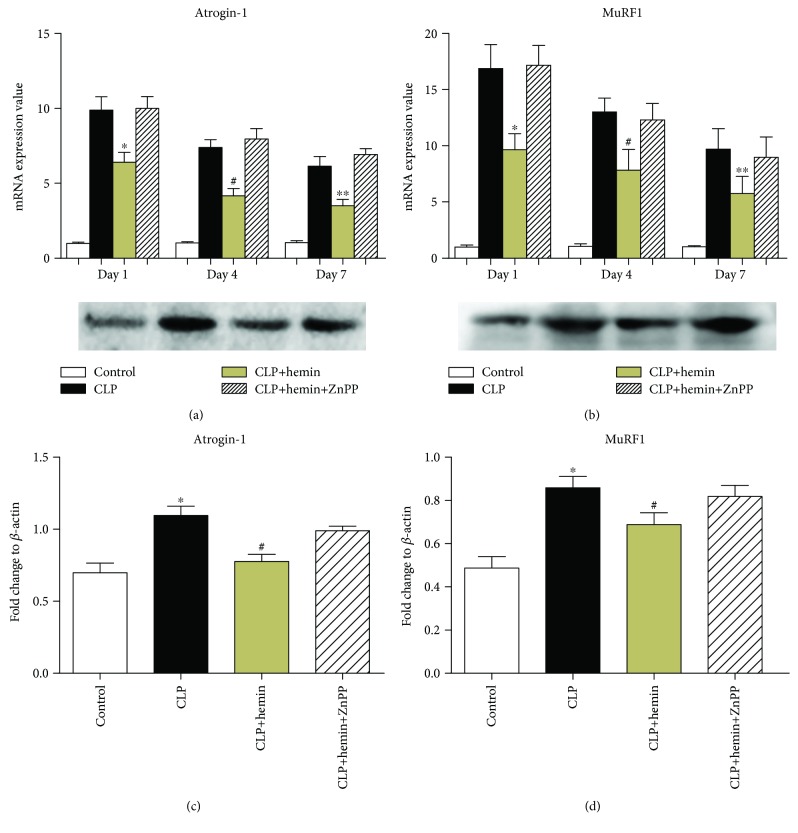
The levels of CLP-induced muscle atrophy markers were elevated, and HO-1 suppressed the mRNA and protein expression of atrogin-1 and MuRF1. (a) HO-1 inhibited the mRNA expression of atrogin-1 at 1, 4, and 7 days after surgery (^∗^*p* < 0.01 vs. CLP at 1 day, ^#^*p* < 0.05 vs. CLP at 4 days, ^∗∗^*p* < 0.01 vs. CLP; *n* = 6). (b) HO-1 suppressed the mRNA expression of MuRF1 during sepsis at 1, 4, and 7 days after surgery (^∗^*p* < 0.01 vs. CLP at 1 day, ^#^*p* < 0.01 vs. CLP at 4 days, ^∗∗^*p* < 0.01 vs. CLP at 7 days; *n* = 6). (c, d) Western blot analysis of levels of the atrogin-1 and MuRF1 proteins. Results of the corresponding semiquantitative analysis of levels of the HO-1 protein based on the optical density measured using the ImageJ software; the data are presented as means ± SEM and are representative of three separate experiments (^∗^*p* < 0.01 vs. control, ^#^*p* < 0.01 vs. CLP). The protein expression of atrogin-1 and MuRF1 was upregulated in the CLP group. Hemin suppressed the protein expression of these two ligases; however, ZnPP administration abrogated the protective effects of hemin (1 day after surgery).

Atrogin-1 and MuRF1 function as muscle-specific ubiquitin E3 ligases that tag proteins destined for ubiquitin-proteasomal proteolysis; thus, they are two of the most important genes upregulated in the process of muscle atrophy. Figures 6(a) and 6(b) show that the mRNA expression levels of atrogin-1 and MuRF1 were significantly upregulated in the TA tissues from CLP mice, especially at 1 day and 4 days after surgery. The enhanced expression of HO-1 induced by hemin reduced the expression of atrogin-1 and MuRF1 in the hemin group compared with that in the CLP group. The expression of the two ubiquitin E3 ligases was higher in the ZnPP group than in the HO-1 group. As shown in Figures 6(c) and 6(d), the protein expression of atrogin-1 and MuRF1 was elevated in the CLP group compared to the observed levels in the control group. Pretreatment with hemin dampened the protein expression of these two E3 ligases. However, in the ZnPP group, the protein expression of atrogin-1 and MuRF1 was upregulated.

## 4. Discussion

In our study, we used the CLP mouse model of sepsis to investigate the protective role of hemin in skeletal muscle wasting. Mice in the CLP group showed reduced muscle force and severe pathological atrophy of myosin fibers. Hemin succeeded in promoting the expression of HO-1 in CLP mice. Compared with mice in the CLP group, hemin-pretreated mice showed an apparent improvement in skeletal muscle force and ameliorated muscle atrophy.

Many factors, such as aging, weightlessness, immobilization, corticosteroids, hyperglycemia, and sepsis, may cause loss of muscle mass and muscle atrophy [23]. Previous studies have shown that early initiation of physical rehabilitation and minimization of deep sedation may prevent disuse atrophy and muscle weakness [24]. Muscle atrophy in critically ill patients could also be attenuated by intensive insulin therapy [25], suggesting that glycemia control is an effective way to protect against muscle proteolysis.

The mechanisms of muscle breakdown are complicated. The ubiquitin-proteasome system plays a primary role in sepsis-induced muscle atrophy, which suggests that ubiquitin ligases may be involved in the development of muscle atrophy during sepsis [26]. There is persistent loss of myosin heavy chain (MyHC) in the skeletal muscle of severe critically ill patients that manifests shortly after ICU admission [27]. MyHC and many other myofibrillar proteins (such as myosin-binding proteins) were regarded as MuRF1 substrates [28]. The atrogin-1 substrates recognized thus far include MyoD, which is thought to control myoblast identity and differentiation [29]. As a result, atrogin-1 and MuRF1, the two muscle-specific ubiquitin ligases, have become potential therapeutic targets [30]. Sepsis, systemic inflammatory response syndrome, and multiple organ failure seem to play a crucial role in leading to muscle atrophy [31]. Proinflammatory cytokines such as TNF-*α* and IL-6 influence the blood-nerve barrier and promote endothelial cell leukocyte activation, which damages the axon [32]; this impaired nerve function may contribute to muscle atrophy. TNF-*α*, IL-6, and other proinflammatory cytokines can also initiate network cascades that activate the FoxO family, the members of which stimulate the expression of MuRF1 and atrogin-1 [30, 33].

HO-1, the rate-limiting enzyme in heme degradation, was reported to confer cytoprotection against oxidative stress and inflammation in several animal models [34]. The major function of HO-1 is to catabolize the oxidative degradation of heme into biliverdin, CO, and free iron. Biliverdin is an effective antioxidant that eliminates peroxyl radicals, and CO exhibits strongly antiapoptotic and anti-inflammatory activities [35]. Previous in vivo and in vitro studies have demonstrated that HO-1 reduces the production of proinflammatory cytokines, including TNF-*α* and IL-6, and inhibits the activation of NF-*κ*B and MAPK signaling during sepsis [36]. HO-1 may prevent liver fibrosis and alleviate lung pathological injury by suppressing NF-*κ*B signaling pathways [37, 38]. Many factors, such as hyperoxia, hypoxia, endotoxins, and nitric oxide, can promote elevated HO-1 expression [39]. In our study, we found that during the early stage of sepsis (24 hours after CLP surgery), there was an obvious increase in HO-1 levels in the CLP group. Hemin, a substrate of HO-1, can enhance the activation of HO-1. In our experiments, we used hemin as an inducer of HO-1 and found that hemin can stimulate HO-1 activation. To identify whether the protective function rooted in HO-1 was mediated by hemin or by other subjects, we included a CLP mouse group treated with ZnPP, an inhibitor of HO-1 that can suppress the enzyme activity of HO-1. We found that mice in the ZnPP group showed no improvements in muscle condition; thus, the protective effects were due to HO-1 but not hemin or other related constituents. Interestingly, we found that ZnPP does not influence the expression of HO-1 but can inhibit its activity, indicating that the protective function of HO-1 may depend on its metabolites.

In our experiments, we found that high levels of HO-1 suppress the gene expression of atrogin-1 and MuRF1, suggesting that HO-1 inhibits muscle fiber atrophy partly by downregulating ubiquitin ligase activation. The CLP model mice showed high levels of TNF-*α* and IL-6 expression in the skeletal muscle. HO-1 suppressed the levels of these proinflammatory cytokines, indicating that HO-1 may inhibit muscle atrophy partly via its protective role against CIP. Oxidative stress is an important contributor to the etiology of skeletal muscle dysfunction. Appropriate levels of oxidants are required for normal cell adaptation and function, and high levels of ROS negatively affect the structure and function of macromolecules such as proteins, DNA, and lipids [40]. High levels of ROS may also activate proteolytic systems, thus causing enhanced protein breakdown in skeletal muscles, which results in enhanced MuRF1 levels leading to muscle atrophy [41]. Our study shows that HO-1-treated mice exhibited a high level of SOD activity and relatively low MDA levels, indicating that HO-1 exerted a protective role in muscle atrophy partly via its antioxidative function.

Regarding the experimental group design, we considered that after surgery, mice in the CLP group and control group may have different appetites, and food intake can also cause differences in muscle mass. To address this, we offered the same quantity of food to all the groups (the exact food quantity was designated based on the results of our preliminary experiments). There are still some questions our experiments did not elucidate. First, we only explored the protective mechanism of HO-1 in the proteolysis process of muscle atrophy, but we did not investigate whether HO-1 can promote muscle protein synthesis. Second, a recent finding showed that hemin exhibited cytotoxicity in colon-derived epithelial cells in a dose- and time-dependent manner. Hemin can downregulate the expression of the 18 kDa translocator protein, which contributes to cell proliferation [42]. Therefore, we must explore other functions of hemin aside from its protective role against muscle atrophy. There are also some limitations in our experiments. First, the experiments were done in both slow-twitch and fast-twitch muscles. It is then difficult to address whether the effect of hemin is specific or not. Second, although the CLP-induced muscle weakness is evident, we did not perform objective measurement to illustrate the issue.

## 5. Conclusion

In conclusion, our findings showed that hemin may promote the elevated expression of HO-1, which exerts a protective function against sepsis-induced skeletal muscle wasting. HO-1 can reduce muscle proteolysis partly by inhibiting the expression of proinflammatory cytokines and muscle-specific ubiquitin ligases. HO-1 can also exert a protective role by suppressing ROS activation in the skeletal muscle. Therefore, hemin might be a new treatment target against sepsis-induced skeletal muscle atrophy.

## Data Availability

The data including muscle mass loss, soleus weight of mice, protein breakdown rates, H&E stating of TA muscles, expression of HO-1, HO-1 activity, Western blot, expression of TNF-alpha and IL-6, MDA, SOD, and expression of atrogin-1 and MuRF1 used to support the findings of this study are included within the article.
